# Effects of heart valve prostheses on phase contrast flow measurements in Cardiovascular Magnetic Resonance – a phantom study

**DOI:** 10.1186/s12968-016-0319-1

**Published:** 2017-01-16

**Authors:** Johanna Richau, Matthias A. Dieringer, Julius Traber, Florian von Knobelsdorff-Brenkenhoff, Andreas Greiser, Carsten Schwenke, Jeanette Schulz-Menger

**Affiliations:** 1 Working Group on Cardiovascular Magnetic Resonance Imaging, Experimental and Clinical Research Center, joint cooperation of the Max-Delbrück-Centrum and Charité -Medical University Berlin, Berlin, Germany; 2 HELIOS Klinikum Berlin-Buch, Department of Cardiology and Nephrology, Berlin, Germany; 3 Siemens Healthcare GmbH, Erlangen, Germany; 4 Department of Cardiology, Clinic Agatharied, Ludwig-Maximilians-University Munich, Hausham, Germany; 5 SCO:SSiS, Berlin, Germany

**Keywords:** Cardiovascular magnetic resonance, Phase contrast, Flow phantom, Valve prosthesis, Artefacts

## Abstract

**Background:**

Cardiovascular Magnetic Resonance is often used to evaluate patients after heart valve replacement. This study systematically analyses the influence of heart valve prostheses on phase contrast measurements in a phantom trial.

**Methods:**

Two biological and one mechanical aortic valve prostheses were integrated in a flow phantom. B_0_ maps and phase contrast measurements were acquired at a 1.5 T MR scanner using conventional gradient-echo sequences in predefined distances to the prostheses. Results were compared to measurements with a synthetic metal-free aortic valve.

**Results:**

The flow results at the level of the prosthesis differed significantly from the reference flow acquired before the level of the prosthesis. The maximum flow miscalculation was 154 ml/s for one of the biological prostheses and 140 ml/s for the mechanical prosthesis. Measurements with the synthetic aortic valve did not show significant deviations. Flow values measured approximately 20 mm distal to the level of the prosthesis agreed with the reference flow for all tested all prostheses.

**Conclusions:**

The tested heart valve prostheses lead to a significant deviation of the measured flow rates compared to a reference. A distance of 20 mm was effective in our setting to avoid this influence.

## Background

Approximately 280.000 heart valve prostheses are implanted worldwide each year; approximately 50% of them are mechanical and 50% bioprosthetic. Due to the medical development and increasing life expectancy, the number of surgeries and interventions is expected to rise [1, 2]. Currently, surgical aortic valve replacement with biological or mechanical prostheses is the standard of care in most cases of aortic stenosis [3, 4]. In patients with high risk to the surgical procedure, transcatheter aortic valve implantation (TAVI) can be considered [3, 5, 6]. Most prostheses have metal compounds in varying amounts and compositions [7].

After valve replacement, the evaluation of prosthetic function and potential prosthesis-related complications is essential [1, 6, 8]. The transthoracic echocardiography (TTE) is the first-line modality for this assessment [9, 10], but the validity of TTE may be limited due to the physical condition of the patient, especially in a postoperative setting. Furthermore, TTE is known to have a high observer dependency [11–13]. Cardiovascular Magnetic Resonance (CMR) is independent of the patient’s anatomy and less observer dependent [13]. Flow measurement with phase contrast (PC) sequences in CMR is used for evaluation and quantification of heart valve disease; it is regarded as robust and valid [11, 14, 15]. In biological valve prostheses, the direct assessment of the orifice area and the analysis of the flow pattern using CMR have already been demonstrated [16–18].

In patients with heart valve prostheses, the metallic compounds often evoke image artefacts, such as phase alterations, image distortion, or even signal loss due to the local distortion of the magnetic field [19].

The purpose of this study is to investigate, whether metal-containing heart valve prostheses have influence on PC based flow measurements.

## Methods

The study was performed with a clinical 1.5 T MR system (MAGNETOM Avanto, Siemens Healthcare GmbH, Erlangen, Germany) equipped with a 6-channel body array coil in combination with a spine array coil.

### Phantom setup

We used a custom-built closed-circuit flow phantom [20]. It consists of a semi-rigid tube system with a pump generating adjustable constant flow and an acrylic pipe resembling the dimensions of the native aortic root. This acrylic pipe could hold different prostheses. The system was filled with a mixture of glycerol (40%) and water (60%) to approximate blood viscosity and relaxation times.

**Fig. 1:**
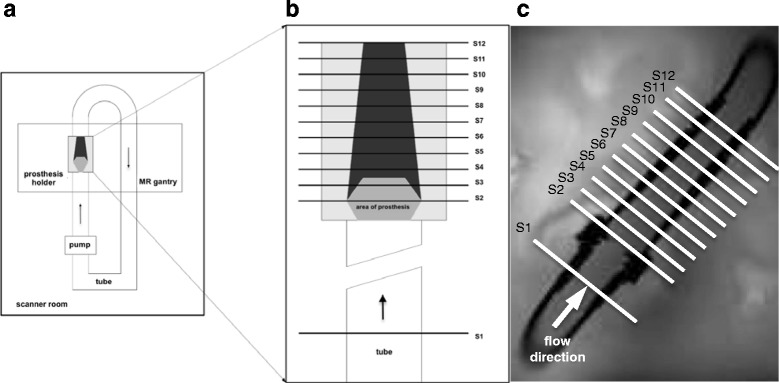
Setting of the MR flow phantom for in-vitro assessment of heart valve prostheses. **a**. Scheme of the experimental setting showing the closed circuit flow phantom as placed in the scanner. **b.** Scheme of the acrylic pipe and positioning of the measurement slices (S1-S12). **c**. Coronal magnitude image of the acrylic pipe, proximal and distal parts of the tube system and positioning of the measurement slices. The arrow demonstrates the direction of flow

The flow phantom was inserted in a water-filled container. The acrylic pipe was placed in the coronal plane 45° oblique between the head-foot and left-right axes to imitate the alignment of the ascending aorta in-vivo. The setting is illustrated in Fig. 1.

To assess background phase errors, all imaging slices were obtained with the phase contrast sequence while the pump was switched off. To avoid unintended flow in the water-filled container, we performed the first scan after 15 min rest following each table movement. Flow measurements in the phantom were performed with the synthetic metal-free aortic valve, the prosthesis I, II and III. Subsequently, the measurements were repeated in the same order with the pump switched on at fixed submaximal power. The flow output varied due to the different orifice areas of the valves.

#### Synthetic aortic valve

**Fig. 2:**
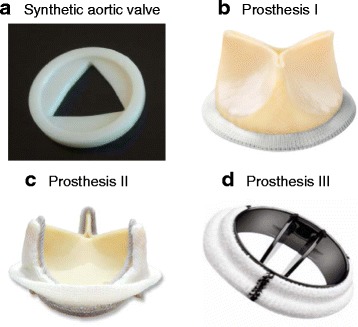
Objects investigated in flow phantom. **a**. Metal-free aortic valve shaped inlay, containing solely plastics. **b**. Biological aortic valve prosthesis, containing metal-stent. **c**. Biological aortic valve prosthesis, containing metal-stent. **d**. Mechanical aortic valve prosthesis, containing carbon

A 3D-printed aortic valve shaped synthetic inlay (Fig. 2) served as the metal-free model. It approximated the prostheses with an orifice area of about 2.0 cm^2^ (Table 1). Flow measurements were compared between the metal-free aortic valve model and the metal-containing prostheses to identify metal-related influences.

**Table 1:** Detailed schedule of material components

Investigated objects	Material components, percentage of metal elements, orifice area
Synthetic aortic valve	Acrylonitrile-Butadiene-Styrene copolymer (ABS plus), 3D printed
	Orifice area: 2.0 cm^2^
Prosthesis I	Stent: polyoxymethylene, covered with polyester cloth
	Annulus: tungsten, silicon
	Leaflet: glutaraldehyde fixated bovine pericardium
	Orifice area: 2.0 cm^2^
Prosthesis II	Stent: corrosion-resistant Elgiloy (cobalt 40%, chromium 20%, nickel 15%, molybdenum 7%, manganese 2%, carbon <0.10%, beryllium <0.10%, iron 5.8%)
	Leaflet: bovine pericadium
	Orifice area: 1.8 cm^2^
Prosthesis III	Orifice: pyrolythic carbon
	Leaflets: pyrolytic carbon graphite coated and tungsten (20%) impregnated
	Annulus: pyrolytic carbon, velour polyester, titanium, coated with Hemashield conduit (double velour polyester collagen impregnated)
	Orifice area: 1.55 cm^2^

#### Prostheses

We investigated three metal-containing aortic valve prostheses from different manufacturers (Fig. 2). Prosthetic sizes were chosen to yield similar orifice areas.

Prostheses I and II are biological valve prostheses consisting of bovine pericardium mounted on a stent. Prosthesis III is a mechanical bileaflet valve.

The details of material composition based on manufacture’s data are listed in Table 1. All investigated prosthetic types are in clinical use for surgical aortic valve replacement and are regarded as MRI-conditional [10, 21–23].

### In-vivo example

To illustrate the effect in vivo, we have added single cases with identical types of aortic valve prostheses and a healthy volunteer. Echocardiography was performed within last 6 months and revealed an adequate valve function as well as a non-dilated ascending aorta.

Additionally one healthy volunteer was scanned as an in-vivo control reflecting the synthetic aortic valve of the phantom trial.

The protocol was similar to the phantom study.

### MR measurements

The following scan protocol was applied in all prostheses. Localizer scans were performed to locate the aorta model. B_0_ maps were acquired in the coronal long axis of the model. Flow measurements were performed in 12 slices transversal to the flow direction.

#### B_0_ mapping

B_0_ maps in coronal planes were acquired in the absence of flow to reveal local magnetic field inhomogeneity. A multi-echo gradient echo sequence with a total of 5 echoes was used. Sequence parameters were: repetition time TR = 50.00 ms, echo time TE_1_ = 3.86 ms, TE_2_ = 9.12 ms, TE_3_ = 14.38 ms, TE_4_ = 19.64 ms, TE_5_ = 24.90 ms, slice thickness 5 mm + 1 mm gap, flip angle 15°, voxel size 2.0 × 2.0 × 5.0 mm^3^.

B_0_ maps were computed using MatLab (The MathWorks, Natick, MA, USA).

**Fig. 3:**
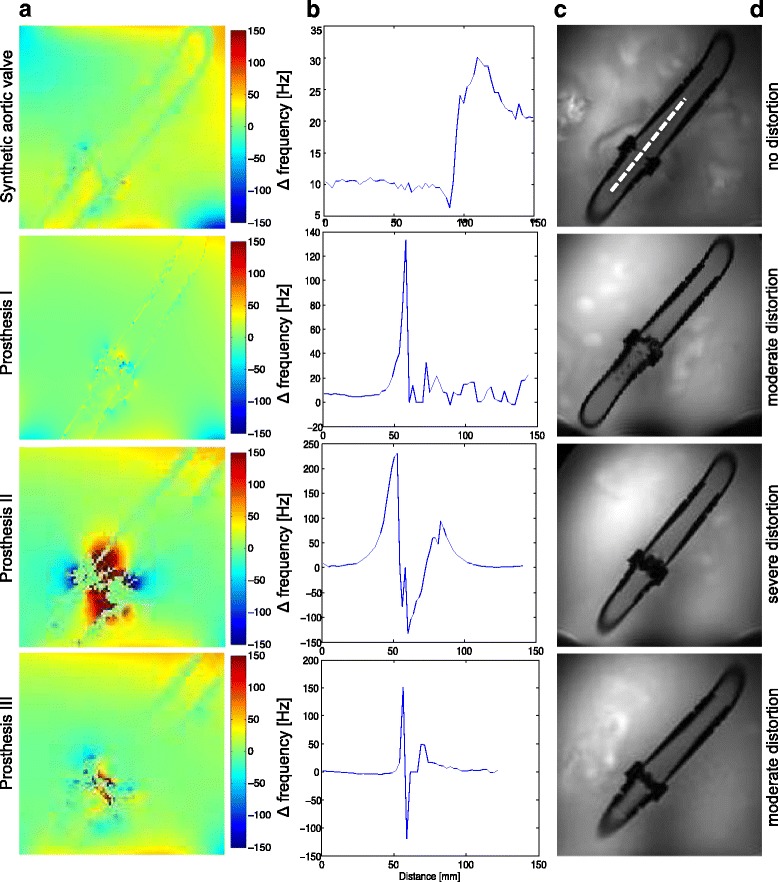
Results of B_0_ mapping. **a**. Coronal images of B_0_ maps, coloured scale in Hertz. **b**. Corresponding profile plots showing the quantitative magnetic field distortion from B_0_ in Hertz. **c**. Corresponding coronal magnitude images. White dashed line top right: Intraluminal positioning of slice for generating profile plots in MATLAB. **d**. Description of the magnetic field distortions according to quantitative score

Quantitative B_0_ profile plots along the long axis of the flow phantom were generated. Therefore, an intraluminal long axis slice was set through the level of the prosthesis (demonstrated in Fig. 3 *top right*).

To objectively assess the visual impressions from magnitude images and B_0_ maps, we used a quantitative score to describe the local magnetic field distortion. The score was defined by quantifying the distortions of the local magnetic field in Hertz (Hz) derived from the profile plots seen in Fig. 3. Magnetic field distortions from B_0_ of less than 50 Hz were graded as ‘none’, from 51 to 150 Hz as ‘mild’, 151 to 250 Hz as ‘moderate’ and more than 250 Hz as ‘severe’.

#### PC flow measurement

A conventional segmented gradient-echo-based PC cine sequence was applied. Sequence parameters were: temporal resolution 47.7 ms, TE 2.3 ms, TR 5.9 ms, slice thickness 5.5 mm + 1.1 mm gap, flip angle 30°, inplane resolution 1.9 × 1.9 mm^2^, GRAPPA reduction factor 2.

To maintain sequence parameters consistent throughout the measurements, but also to avoid possible phase aliasing due to the different maximum flow velocities in the prostheses, velocity encoding was set to 500 cm/s for all measurements.

A 60-beats-per-minute ECG simulator was used as acquisition trigger.

PC images were acquired at 12 positions: Slice 1 (S1) was set perpendicular to the tube and 40 mm proximal to the level of the prosthesis. This slice was defined as reference flow level, where constant laminar flow was assumed. S2 was set at the level of the prosthesis and was positioned to the isocenter of the scanner. Ten more positions were defined (S3-S12) based on slice thickness 5.5 mm +1.1 mm gap distal to the level of the prosthesis (see Fig. 1). S1 and S3 to 12 were not acquired in the isocenter to avoid table movement and therefore fluid motion in the water-basket.

Due to anatomical reasons we could not define a reference measurement point in-vivo similar to the phantom. So the in-vivo measurements start at the area of the aortic valve with slice 2 (S2) and amount 11 measurement positions.

PC measurements were repeated three times per position and flow results were averaged.

PC images were analysed using cvi42 Version 4.1.5 (Circle Cardiovascular Imaging, Calgary, Canada). Segmentation of the tube was done in the magnitude image. Contours were automatically propagated into all temporal phases of the phase image and manually adapted if necessary.

### Statistical analysis

Calculations were performed using SAS 9.2 (SAS Institute Inc., Cary, NC, USA). Figures were prepared using R Version 3.2.1 [24] with packages ggplot2 [25] and RColorBrewer [26]. Least-square mean flow deviations from reference flow were calculated along with 95% confidence intervals (CI) in a statistical linear mixed model with the flow at the reference level (S1) as co-factor. The sensitivity analyses based on non-parametric models supported the outcomes of the linear mixed model and are not further displayed. Other factors were the distance and the type of prosthesis. Due to the small sample size, the statistical linear mixed model was repeated based on ranks to account for any non-normality of the data as sensitivity analysis to check for the robustness of the assumption of normality. The flow deviations were presented graphically per type of prosthesis. In addition, the distance to the level of the prosthesis was displayed along with equivalence limits of ±15% and ±20%. The equivalence limits were calculated as mean flow at the reference level ± the defined percentage limit of clinical irrelevance. As no validated or established limit of equivalence is published, two reasonable limits are reported here.

Significance in this setting is defined as a deviation of the flow values from reference flow (S1) of less than 15% or 20% respectively (range of equivalence). Positions in that range are described as significant, i.e. showed equivalence referred to the reference flow. Conversely no equivalence can be concluded for values out of this range. Convergence of flow values is defined as mean flow difference of less than 15% to reference flow and nearly identical flow values beyond that distance.

## Results

All PC image series were successfully acquired and evaluable.

### B_0_ mapping

**Fig. 4:**
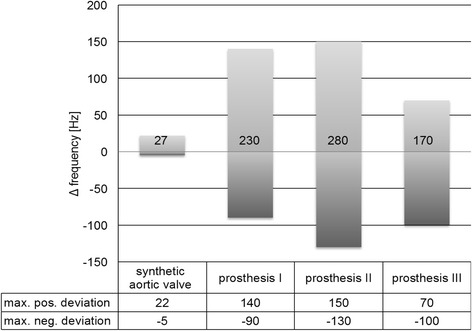
Quantitative aberration from B_0_ magnetic field in all investigated objects. Deviation in Hertz (Hz) derived from B_0_ maps and profile plots. Corresponding table showing positive and negative maximum of field aberration in Hz over the whole investigated distance. The numbers within the bars indicate the absolute range of deviation in Hz, as it is used for the quantitative score

The B_0_ maps in Fig. 3 demonstrate that depending on the type of valve, the local magnetic field distortions varied. In the synthetic aortic valve, no relevant distortion was detected. In contrast, the B_0_ maps of the prostheses showed moderate and severe distortions. The quantitative aberration from B_0_ magnetic field as an expression of distortion is shown in Fig. 4. It was 27 Hz in the synthetic aortic valve, which was classified as not relevant according to the score. In contrast, the aberration was 230 Hz in biological prosthesis I and 170 Hz in the mechanical valve III, which were classified both as moderate. In biological prosthesis II, the aberration was 280 Hz, which was classified as severe distortion.

### PC flow measurement - flow phantom

**Fig. 5:**
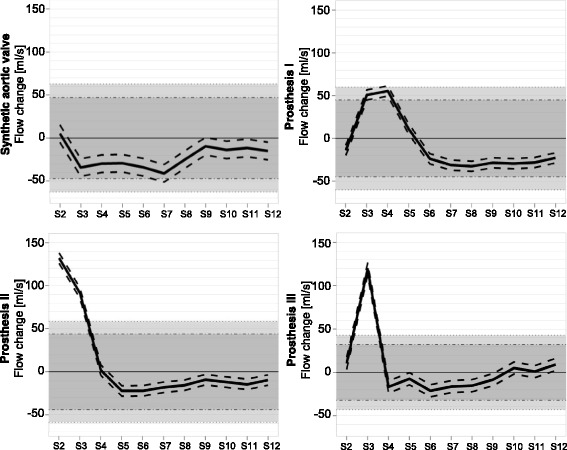
Flow deviation. Deviation of the assessed flow from reference flow over distance. Dark grey: range of equivalence 15%. Light grey: range of equivalence 20%. Black dashed lines: 95% confidence interval

As seen in Table 2 and Fig. 5, the synthetic aortic valve showed a maximum absolute flow deviation over the whole distance of 46 ml/s, corresponding to 15% of the baseline flow. In bioprosthesis I, the flow deviation amounted 88 ml/s, corresponding to 29% of the baseline flow. Bioprosthesis II showed 154 ml/s flow deviation, corresponding to 53% of the baseline flow. In the mechanical prosthesis, a flow deviation of 140 ml/s was observed, corresponding to 35% of the baseline flow. There was a convergence to baseline flow after 19.8 mm (3 slice distances) distal to the bioprosthesis I, in bioprosthesis II and the mechanical prosthesis after 13.2 mm (2 slice distances).

**Table 2:** Results of Phase contrast-based flow measurement

	Synthetic aortic valve	Prosthesis I	Prosthesis II	Prosthesis III
a. Peaks of flow deviation (mean min/max) [ml/s]	5.28 (−4.92 to 15.48) at S2 to −41.03 (−51.23 to −30.83) at S7	55.47 (49.54 to 61.39) at S4 to −32.41 (−38.34 to −26.49) at S8	132.20 (126.13 to 138.27) at S2 to - 22.20 (− 28.27 to −16.13) at S5	119.13 (112.14 to 126.12) at S3 to - 21.15 (− 28.14 to - 14.16) at S6
b. Absolute flow variation [ml/s]	46	88	154	140
Percentage according to baseline [%]	15	29	53	35
c. Significance within 15% range of equivalence [ml/s]	all positions, except S7 +/−47.08	all positions, except S3 & S4 +/−44.91	all positions, except S2 & S3 +/−43.94	all positions, except S3 +/−32.20
Significance within 20% range of equivalence [ml/s]	all positions +/−62.77	all positions, except S4 +/−59.87	all positions, except S2 & S3 +/−58.59	all positions, except S3 +/−42.94
d. Convergence to reference flow [mm]		19.8 (S5)	13.2 (S4)	13.2 (S4)

a. Min. & max. deviation of flow values from reference flow (S1) & their local attribution (peak to peak), 95% confidence interval bracketed

b. Absolute flow variation between the two peaks. Percentage of flow variation according to the absolute flow values at S1

c. Significances for all measured slices within two ranges of equivalence (15% and 20%)

d. Convergence of flow values: mean flow deviation of less than 15% to reference flow, nearly identical flow values beyond that distance

S1-12 = slice 1 to 12, positions of image acquisition in flow phantom

### PC flow measurement - in-vivo example

**Fig. 6:**
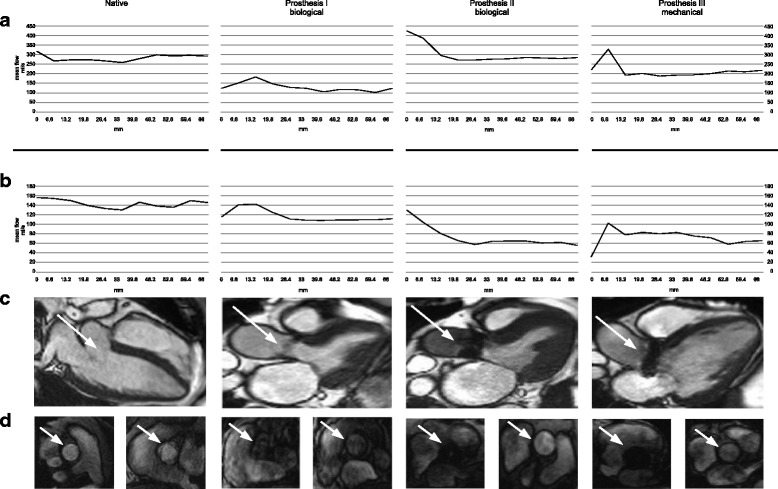
PC flow measurements in-vitro and in-vivo including a synthetic aortic valve model, two biological and one mechanical aortic valve prosthesis. **a**. Flow measured in-vitro over the distance to the valve. **b.** Flow measured in-vivo over the distance to the valve. **c** In-vivo: Long axis view (Cine-imaging, steady state free precession) showing the left ventricular outflow tract and the position of the aortic prosthesis (*white arrow*). **d** In-vivo: cine imaging of the aortic valve location (left) and ascending aorta in the area of the suspected restored flow 26.4 mm distal to the aortic valve (right) for each subject (columns)

As seen in Fig. 6b) the volunteer showed a maximum absolute flow deviation over the whole distance of around 18 ml/s with a maximum mean flow of 154 ml/s in the area of the native aortic valve (S2 compared to phantom) and a minimum mean flow of 136 ml/s 33 mm distal to the area of the aortic valve (S7 compared to phantom).

In bioprosthesis I, the flow deviation amounted 28 ml/s with a maximum mean flow of 71 ml/s 13.2 mm distal to the area of the prosthesis (S4 compared to phantom) and a minimum mean flow of 42 ml/s 59.4 mm distal to the area of the aortic valve (S11 compared to phantom).

Bioprosthesis II showed 65 ml/s flow deviation with a maximum mean flow of 123 ml/s in the area of prosthesis (S2 compared to phantom) and a minimum mean flow of 59 ml/s 26.4 mm distal to the area of the aortic valve (S6 compared to phantom).

In the mechanical prosthesis III a flow deviation of 64 ml/s was observed with a maximum mean flow of 100 ml/s 6.6 mm distal to the prosthesis (S3 compared to phantom) and a minimum mean flow of 37 ml/s in the area of the prosthesis (S1 compared to phantom).

The flow volume curves of patient and flow phantom are showing a similar shape as seen in Fig. 6. A normalization of flow volume could be identified approximately 26 mm distal to the prostheses level in all in-vivo tests. At this position no artefacts could be identified anymore in the corresponding anatomical images.

The biological valve prosthesis II showed the most severe artefacts and flow miscalculation, which meets also the result of the in-vitro trial. A comparison between in-vivo cine imaging (Fig. 6c) and d)) and in-vitro magnitude pictures (Fig. 3) shows the accordance clearly.

### Background offset error detection in the absence of flow

Statistical analyses of the range of equivalence were not meaningful for the measurements without flow generated by the pump, as the reference flow values were too low. In this case, significance is defined as a relevant deviation from zero flow in ml/s with a two-sided p-value below or equal 0.05.

**Fig. 7:**
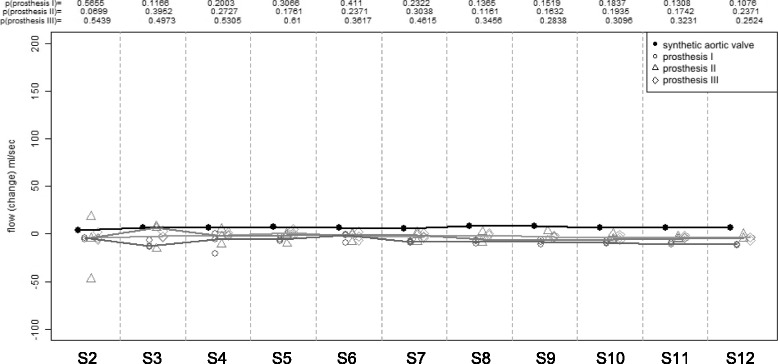
Background offset error detection. Measurements without flow. Deviation of estimated flow from reference flow over the entire distance in all investigated objects. S2-12 = positions of image acquisition in flow phantom

Flow measurements without flow revealed no differences between reference level and the slices distal to the prosthesis in all investigated objects. As depicted in Fig. 7, there is no significant deviation of flow values over the entire distance (*p* > 0.05), meaning no relevant background offset errors.

## Discussion

This phantom study investigated the influence of three different metal containing heart valve prostheses on PC flow measurements at 1.5 T. The main finding is that the flow measurements were corrupted in the vicinity of the tested valve prostheses. The extent of flow miscalculation depended on the composition of the prosthesis and on the distance of the measurement slice to the prosthesis. In contrast, PC measurements even in close proximity to a synthetic metal-free aortic valve model or a native aortic valve yielded nearly constant flow values.

PC measurements are expected to be B_0_-insensitive, as two images containing the same flow-unrelated phase errors are subtracted from each other [27]. In this study, we could reproduce this finding in measurements where no flow was present – notwithstanding all tested metal containing prostheses markedly altered the local magnetic field. Beyond that, imaging and flow encoding gradients induce eddy currents in the metal stent that evoke superimposing local magnetic field distortions.

In case of no flow, i.e. the pump was switched off, it could be shown that the phase contrast measurement yielded a net phase of zero after subtraction of the flow-compensated from the flow-encoded measurement even in the presence of these additional local magnetic field distortions.

In case of flow, however, the results showed that in the proximity of the metal stented prostheses severe flow measurement errors occurred. As for the static magnetic field related distortions and the imaging gradients related distortions, the flow-encoded measurement and the flow-compensated measurement experience identical field alterations. However, as the flow-encoding gradient scheme and the flow-compensating gradient scheme are dissimilar, these gradient schemes also induce different local and temporal magnetic field distortions. Analysis of the B_0_ maps in the proximity of the heart valve prostheses revealed that the severity of local magnetic field inhomogeneity was coincident with the severity of flow miscalculations. Coherence between the extent of the flow miscalculation and the material composition of the prosthesis seems to be likely as different material compositions inherit different magnetic susceptibilities and thus induce different magnetic field distortions. Analogous to the fact that the interaction with more susceptible material leads to stronger B_0_ inhomogeneity, the interaction of the gradients with the more susceptible material also leads to stronger net phase errors. Beyond that, also flow velocity influences the amount of additionally accumulated phase and thus the extent of flow miscalculation.

The strongest local magnetic field inhomogeneity and maximum flow miscalculation were observed in prosthesis II, with a maximum flow overestimation of around 150 ml/s in phantom, which is 53% deviant to the reference flow. In-vivo around 65 ml/s flow deviation was seen.

Our findings are related to the dedicated material of these prostheses. The influence on the magnetic field depends on the material. The induced local magnetic field distortions have different impact on flow measurements [19, 28].

The impact of heart-valve-prostheses-related magnetic field distortions depended on the distance of the measurement slice to the level of the prostheses. Maximum miscalculation is seen in the immediate proximity of the prostheses. For the investigated prostheses, the flow values converged in-vitro beyond a distance of about 20 mm and in-vivo about 30 mm distal to the level of the prosthesis. We used a range of equivalence of 15% as a marker for significant deviation from reference flow. The background phase offset errors are within this range; so we deduce a flow-related impact of the metal components.

Although it could be shown that heart valve prostheses are MR-safe up to 4.7 T, the magnetically induced electric currents can hamper the diagnostic performance [29–31]. Both biological prostheses cause magnetic field distortions and signal loss to various extents as seen in Fig. 3. Prosthesis I is constructed onto a flexible, tender stent, whereas prosthesis II comprises a semi-rigid, three-base frame. The extent of magnetic field distortion is related to the amount of metal. Another trial demonstrated metal-stented aortic valve prostheses inducing artefacts in CMR in terms of signal loss in the whole left-ventricular outflow tract. In contrast, the stent-less valve prostheses did not cause artefacts [32].

Among the research on heart valve prostheses, magnetic field distortions due to metallic compounds were investigated in several studies, but flow-measurement-related data are rare [33]. In our study, prosthesis II consists of Elgiloy, an alloy recommended for biomedical implants. As seen in Figs. 3 and 4, it evokes local magnetic field distortions. The mechanical prosthesis III, composed mainly of pyrolythic carbon, was deemed to be nonmagnetic, but showed magnetic field distortions.

The interaction between both mechanical and biological valve prostheses and magnetically induced electric currents has been demonstrated [34].

It has already been described that elements from stainless steel cause stronger artefacts than Nitinol-based products [19, 28]. Our in vitro results are supported by single case examples, but have to be evaluated in a larger cohort.

Assessment of valvular prosthesis is of high impact in clinical cardiology and CMR has an increasing influence. Therefore these results could have an impact on applicability of flow quantification in this patient group.

### Strengths and limitations

Our study has the strength that we faced a potential obstacle for clinical decision-making when using phase-contrast techniques in patients with valve prostheses. Our results are limited by the fact that our setting did not include MR-independent flow quantification as a reference. However, the setting has been validated previously [35] and the flow was kept constant to meet the requirements for this study.

In our study, we investigated the behaviour of PC based flow quantification using a flow-encoded and a flow-compensated measurement. As other techniques exist - such as the measurement with two flow-encoded measurements - our findings cannot be generalized for all existing flow measurement techniques in the same manner.

We have investigated three often-used prostheses. Our findings cannot be directly extrapolated to all other types of prosthesis. Especially the recommended distance to achieve correct flow measurements needs careful studies covering all types of prostheses. Further investigation and analysis of all different valve prostheses have to be conducted to address this need.

## Conclusions

Our findings are of clinical interest as they may impact clinical routine. Currently, specific distances are defined for flow measurement of native valves and valve prostheses [36]. But the awareness of magnetic field distortion-related flow miscalculations is limited. However, it can be concluded that flow measurements in the proximity of stented valve prostheses are not reliable.

When performing PC based flow measurements in patients with heart valve replacement, one should be aware of potential flow and volume miscalculation due to prosthesis-related distortions of the magnetic field.

Further investigations regarding other available prostheses are needed, as clinical outcome after valve replacement is based on a patient-matched prosthesis, which has to be determined by reliable measurements.

Further studies are needed to provide guidance for different field strengths and all types of prosthesis.

## Abbreviations

CMR: Cardiovascular magnetic resonance
Hz: Hertz
PC: Phase contrast
S: Slice
T: Tesla
TE: Echo time
TR: Temporal resolution

## References

[1] Pibarot P, Dumesnil JG (2009). Prosthetic heart valves: selection of the optimal prosthesis and long-term management. Circulation.

[2] Hsiao A (2015). Inlet and outlet valve flow and regurgitant volume may be directly and reliably quantified with accelerated, volumetric phase-contrast MRI. J Magn Reson Imaging.

[3] Nishimura RA, et al. 2014 AHA/ACC Guideline for the Management of Patients With Valvular Heart Disease: A Report of the American College of Cardiology/American Heart Association Task Force on Practice Guidelines. Circulation. 2014;129:e521–e643. 10.1161/CIR.0000000000000031 24589853

[4] Lamelas J, Nguyen TC (2015). Minimally Invasive Valve Surgery: When Less Is More. Semin Thorac Cardiovasc Surg.

[5] Smith CR (2011). Transcatheter versus surgical aortic-valve replacement in high-risk patients. N Engl J Med.

[6] Hartlage GR (2014). The role of cardiovascular magnetic resonance in stratifying paravalvular leak severity after transcatheter aortic valve replacement: an observational outcome study. J Cardiovasc Magn Reson.

[7] Walther T (2012). Contemporary management of aortic stenosis: surgical aortic valve replacement remains the gold standard. Heart.

[8] Butchart EG (2005). Recommendations for the management of patients after heart valve surgery. Eur Heart J.

[9] Bach DS (2010). Echo/Doppler evaluation of hemodynamics after aortic valve replacement: principles of interrogation and evaluation of high gradients. JACC Cardiovasc Imaging.

[10] Zoghbi WA (2009). Recommendations for evaluation of prosthetic valves with echocardiography and doppler ultrasound: a report From the American Society of Echocardiography’s Guidelines and Standards Committee and the Task Force on Prosthetic Valves, developed in conjunction with the American College of Cardiology Cardiovascular Imaging Committee, Cardiac Imaging Committee of the American Heart Association, the European Association of Echocardiography, a registered branch of the European Society of Cardiology, the Japanese Society of Echocardiography and the Canadian Society of Echocardiography, endorsed by the American College of Cardiology Foundation, American Heart Association, European Association of Echocardiography, a registered branch of the European Society of Cardiology, the Japanese Society of Echocardiography, and Canadian Society of Echocardiography. J Am Soc Echocardiogr.

[11] Myerson SG (2012). Heart valve disease: investigation by cardiovascular magnetic resonance. J Cardiovasc Magn Reson.

[12] Ciolina F (2015). Aortic valve stenosis: non-invasive preoperative evaluation using 64-slice CT angiography. J Cardiovasc Surg (Torino).

[13] Cawley PJ, Maki JH, Otto CM (2009). Cardiovascular magnetic resonance imaging for valvular heart disease: technique and validation. Circulation.

[14] Markl M, Wallis W, Harloff A (2011). Reproducibility of flow and wall shear stress analysis using flow-sensitive four-dimensional MRI. J Magn Reson Imaging.

[15] von Knobelsdorff-Brenkenhoff F, Trauzeddel RF, Schulz-Menger J. Cardiovascular magnetic resonance in adults with previous cardiovascular surgery. Eur Heart J Cardiovasc Imaging. 2013. 10.1093/ehjci/jet138 23913332

[16] von Knobelsdorff-Brenkenhoff F (2009). Feasibility of cardiovascular magnetic resonance to assess the orifice area of aortic bioprostheses. Circ Cardiovasc Imaging.

[17] von Knobelsdorff-Brenkenhoff F (2010). Assessment of mitral bioprostheses using cardiovascular magnetic resonance. J Cardiovasc Magn Reson.

[18] von Knobelsdorff-Brenkenhoff F, et al. Blood flow characteristics in the ascending aorta after aortic valve replacement-a pilot study using 4D-flow MRI. Int J Cardiol. 2014;170(3):426–33. 10.1016/j.ijcard.2013.11.034 PMC5099073 24315151

[19] Kahlert P (2010). Towards real-time cardiovascular magnetic resonance-guided transarterial aortic valve implantation: in vitro evaluation and modification of existing devices. J Cardiovasc Magn Reson.

[20] von Knobelsdorff-Brenkenhoff F (2011). In vitro assessment of heart valve bioprostheses by cardiovascular magnetic resonance: four-dimensional mapping of flow patterns and orifice area planimetry. Eur J Cardiothorac Surg.

[21] Eichinger WB (2005). Exercise hemodynamics of bovine versus porcine bioprostheses: a prospective randomized comparison of the mosaic and perimount aortic valves. J Thorac Cardiovasc Surg.

[22] Jamieson WR (2010). Hemodynamic performance of mitroflow aortic pericardial bioprosthesis - optimizing management for the small aortic annulus. Thorac Cardiovasc Surg.

[23] Van Nooten GJ (2014). Twenty-year single-center experience with the medtronic open pivot mechanical heart valve. Ann Thorac Surg.

[24] R Core Team. A language and environment for statistical computing. Vienna: The R Foundation for Statistical Computing; 2015.

[25] Wickham H (2009). ggplot2: elegant graphics for data analysis.

[26] Neuwirth E (2014). R ColorBrewer: ColorBrewer Palettes. R package version 1.1-2.

[27] Khodarahmi I (2014). In vitro validation of flow measurement with phase contrast MRI at 3 tesla using stereoscopic particle image velocimetry and stereoscopic particle image velocimetry-based computational fluid dynamics. J Magn Reson Imaging.

[28] Khan SN (2013). Pediatric cardiovascular interventional devices: effect on CMR images at 1.5 and 3 Tesla. J Cardiovasc Magn Reson.

[29] Edwards MB (2005). Assessment of magnetic field (4.7 T) induced forces on prosthetic heart valves and annuloplasty rings. J Magn Reson Imaging.

[30] Condon B, Hadley DM (2000). Potential MR hazard to patients with metallic heart valves: the Lenz effect. J Magn Reson Imaging.

[31] Levine GN (2007). Safety of magnetic resonance imaging in patients with cardiovascular devices: an American Heart Association scientific statement from the Committee on Diagnostic and Interventional Cardiac Catheterization, Council on Clinical Cardiology, and the Council on Cardiovascular Radiology and Intervention: endorsed by the American College of Cardiology Foundation, the North American Society for Cardiac Imaging, and the Society for Cardiovascular Magnetic Resonance. Circulation.

[32] Quail MA (2012). Use of cardiovascular magnetic resonance imaging for TAVR assessment in patients with bioprosthetic aortic valves: comparison with computed tomography. Eur J Radiol.

[33] Kvitting JP (2010). In vitro assessment of flow patterns and turbulence intensity in prosthetic heart valves using generalized phase-contrast MRI. J Magn Reson Imaging.

[34] Edwards MB (2015). In vitro assessment of the Lenz effect on heart valve prostheses at 1.5 T. J Magn Reson Imaging.

[35] Dieringer MA (2012). Design, construction, and evaluation of a dynamic MR compatible cardiac left ventricle model. Med Phys.

[36] O’Brien KR (2009). Aortic valve stenotic area calculation from phase contrast cardiovascular magnetic resonance: the importance of short echo time. J Cardiovasc Magn Reson.

